# Supplementary figures and images for: MiR-34a deficiency enhances nucleic acid sensing and type I IFN signaling in a mouse model of Alzheimer’s disease
Source: Front Immunol. 2026 Feb 24;17:1694824. doi: 10.3389/fimmu.2026.1694824 (PMC12971419; doi:10.3389/fimmu.2026.1694824)

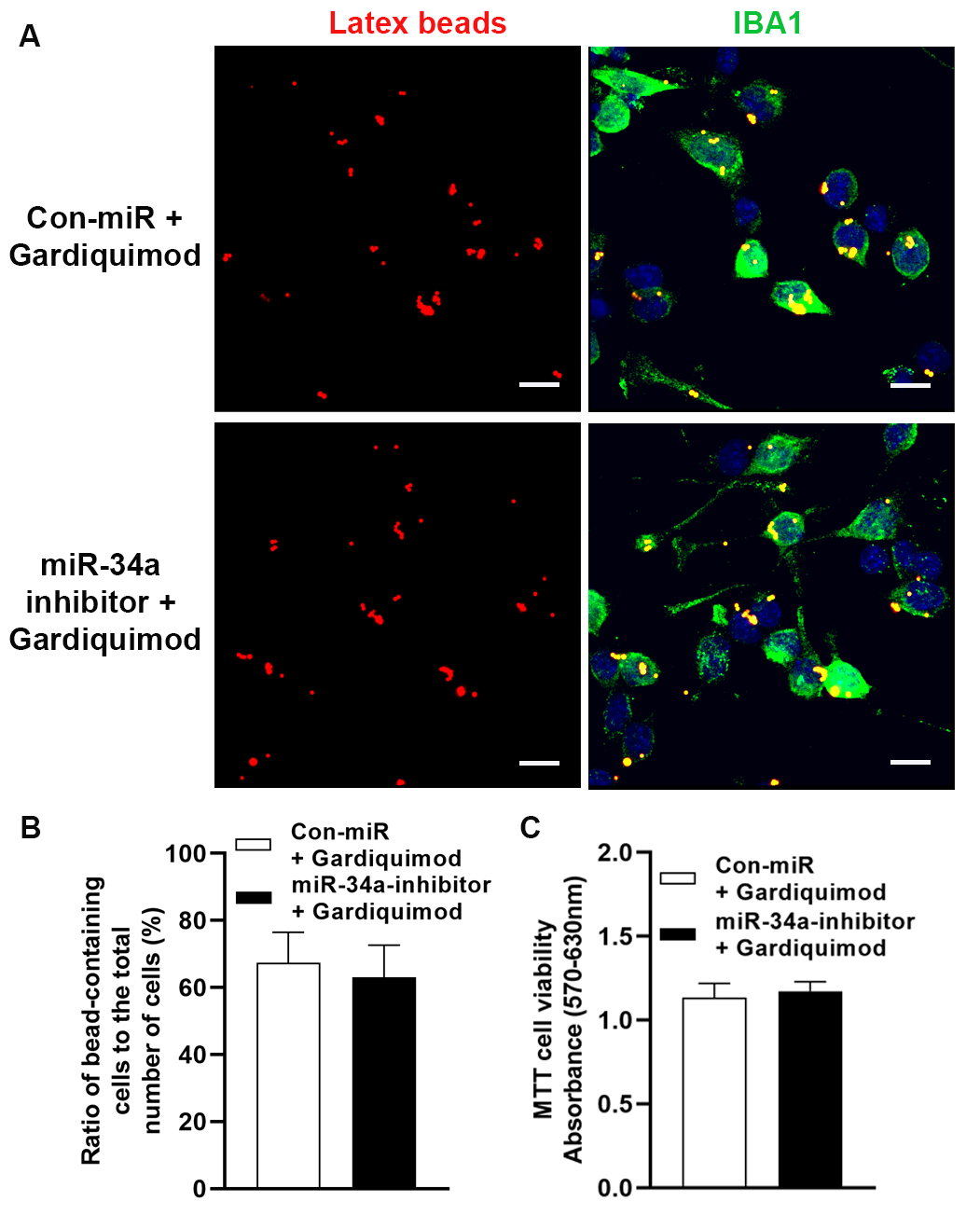

Supplement: Supplementary file 1 [file Image1.tif]

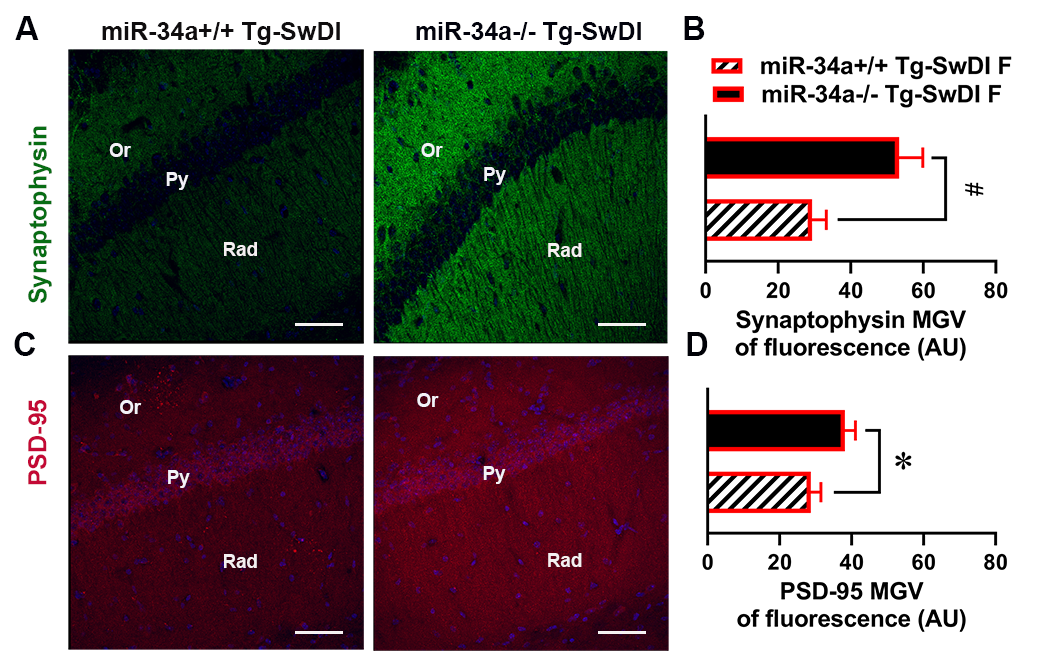

Supplement: Supplementary file 2 [file Image2.tif]
