# Supplementary material for: MiR-34a deficiency enhances nucleic acid sensing and type I IFN signaling in a mouse model of Alzheimer’s disease
Source: Front Immunol. 2026 Feb 24;17:1694824. doi: 10.3389/fimmu.2026.1694824 (PMC12971419; doi:10.3389/fimmu.2026.1694824)
Supplement: Supplementary file 3 [file DataSheet1.docx]

**Supplementary Materials**

1. **Materials and methods**

*1.1 Phagocytosis assay*

BV2 cells were seeded at a density of 8 x 10^4^ in chamber slides (Cat# 12-565-6, Thermo Fisher Scientific) coated with 0.01% poly-D-lysine (Cat #A3890401, Thermo Fisher Scientific) and cultured overnight. Cells were transfected and subsequently treated with 0.2 µg/ml Gardiquimod. After 24 hours of Gardiquimod treatment, the medium was carefully removed and replaced with DMEM containing latex beads (Cat# L2778, Sigma) at a ratio of 10 beads per cell followed by incubation in a cell culture incubator for 6 hours as previously described (1). The bead-containing medium was then gently removed, and the cells were rinsed three times with PBS, followed by fixation with 4% PFA for 30 min at room temperature. BV2 cells were immuno-stained with an IBA1 antibody (1:1000; FUJIFILM Wako Pure Chemical Corporation, Cat# 019-19741, RRID: AB_839504) to outline the cell body. Images were acquired using an Olympus FV3000 confocal microscope (Olympus America Inc.) with consistent settings using a 60× objective.

*1.2 Cell viability measurement*

BV2 cells were seeded in 96-well plates at a density of 1 x 10^4^ cells per well and cultured overnight in in DMEM (ATCC formulation) supplemented with 10% fetal bovine serum (FBS) under standard conditions. Cells were transfected and subsequently treated with Gardiquimod as described in the methods section (2.7 in the manuscript). After 24 hours of Gardiquimod treatment, the medium was carefully removed and 100 µl of Thiazolyl blue tetrazolium bromide (MTT; Cat# 158990050, Thermo Fisher Scientific) at a concentration of 0.5mg/ml in serum-free DMEM (sterilized through a 0.2 µm filter) was added to each well and incubated for 4 hours in a cell culture incubator. The MTT-containing medium was then carefully removed, and 150 µl of DMSO was added to each well. The plate was wrapped in foil and shaken on an orbital shaker for 20 min. Absorbance was measured at 570nm with background subtraction at 630nm using SoftMax Pro5 (Molecular Devices).

*1.3 Immunofluorescence staining and image analysis*

For synaptophysin and PSD-95 staining, antigen retrieval was performed in antigen retrieval buffer (Cat#ab93684, Abcam) at 85 °C for 10 min, followed by cooling to room temperature. Sections were incubated with anti-synaptophysin antibody (1:500; Proteintech, Cat#82900-1-RR, RRID: AB_3670629) and anti-PSD95 antibody (1:300; Proteintech, Cat#20665-1-AP, RRID: AB_2687961) for 48 h at 4 °C. After rinsing (4 x 15 min in TBS), sections were incubated with Alexa Fluo-conjugated anti-Rabbit IgG (1:500; Abcam, Cat# ab150073, RRID: AB_2636877) and Alexa Fluor-conjugated anti-Rabbit IgG (1:500; Invitrogen, Cat# A-21207, RRID: AB_141637) for 2 h at room temperature in the dark. Nuclei were counterstained with DAPI, and sections were mounted using Shandon™ Immu-Mount™ (Thermo Fisher Scientific). Images were acquired using an Olympus FV3000 confocal microscope (Olympus America Inc.) with consistent settings using 40× objectives. The mean gray value (MGV) of PSD-95 and synaptophysin fluorescence staining from the stratum radiatum (Rad) area in CA1 was measured using ImageJ v1.40 (NIH) as previously described(2).

1. **Supplementary Figures and tables**

2.1 Supplementary Figures

**Supplementary Figure 1.** **Effects of miR-34a knockdown on uptake of latex beads and cell viability in Gardiquimod-treated BV2 microglia in vitro.** The uptake of latex beads by BV2 cells is shown in (A). Red indicates the beads and green indicates the Iba1. Scale bars 10 µm. The ratio of bead-containing cells to the total cells is shown in (B). Three areas were randomly captured at 60 x objective from three independent experiments. Cell viability was determined by MTT assay (C). Data from three independent experiments with duplicates were used.

**Supplementary Figure 2. Effects of miR-34a KO (miR-34a^-/-^) on expression of synaptophysin and PSD-95 in the brain**. Brain sections were subjected to immunofluorescence staining using anti-synaptophysin (green) and DAPI (blue) (A), and anti-PSD-95 (red) and DAPI (blue) (C) (Scale bars 50 µm. **Or**: oriens layer, **Py**: pyramidal cell layer, **Rad**: stratum radiatum). The mean gray value (MGV) of fluorescence arbitrary units (AU) in CA1 Rad area was quantified using imageJ software. Data was analyzed by independent T-test. (miR-34a+/+ Tg-SwDI: n=9, miR34a-/- Tg-SwDI: n=10, B for synaptophysin, P = 0.006; and D for PSD-95, P= 0.01, respectively, *P<0.05 and #P< 0.01)

- 1. Supplementary Tables

**Supplementary Table 1**. DEG of experimental mouse groups

**Supplementary Table 2.** GO_BP from Enrichr Analysis

**Supplementary Table 3.** IRM signature genes significantly upregulated in miR-34a-deficient Tg-SwDI mice and potential miR-34a target genes

References

(1) McPherson CA, Kelly-Rajan K, Lefebvre d'Hellencourt C, Harry GJ. High Content Imaging and Quantification of Microglia Phagocytosis In Vitro*. Curr Protoc* (2023) **3**:e638. doi: 10.1002/cpz1.638.

(2) Nowacka A, Borczyk M, Salamian A, Wojtowicz T, Wlodarczyk J, Radwanska K. PSD-95 Serine 73 phosphorylation is not required for induction of NMDA-LTD*. Sci Rep* (2020) **10**:2054–2. doi: 10.1038/s41598-020-58989-2.
